# Evaluation of Chlorophyll-*a* Estimation Approaches Using Iterative Stepwise Elimination Partial Least Squares (ISE-PLS) Regression and Several Traditional Algorithms from Field Hyperspectral Measurements in the Seto Inland Sea, Japan

**DOI:** 10.3390/s18082656

**Published:** 2018-08-13

**Authors:** Zuomin Wang, Yuji Sakuno, Kazuhiko Koike, Shizuka Ohara

**Affiliations:** 1 Graduate School of Engineering, Hiroshima University, 1-4-1 Kagamiyama, Higashi-Hiroshima, Hiroshima 739-8527, Japan; wangzuomin123@gmail.com; 2 Graduate School of Biosphere Science, Hiroshima University, 1-4-4 Kagamiyama, Higashi-Hiroshima, Hiroshima 739-8528, Japan; kazkoike@hiroshima-u.ac.jp (K.K.); oharashizu@hiroshima-u.ac.jp (S.O.)

**Keywords:** water quality, remote sensing, harmful algal bloom, partial least squares regression

## Abstract

Harmful algal blooms (HABs) occur frequently in the Seto Inland Sea, bringing significant economic and environmental losses for the area, which is well known as one of the world’s most productive fisheries. Our objective was to develop a quantitative model using in situ hyperspectral measurements in the Seto Inland Sea to estimate chlorophyll *a* (Chl-*a*) concentration, which is a significant parameter for detecting HABs. We obtained spectra and Chl-*a* data at six stations from 12 ship-based surveys between December 2015 and September 2017. In this study, we used an iterative stepwise elimination partial least squares (ISE-PLS) regression method along with several empirical and semi-analytical methods such as ocean chlorophyll, three-band model, and two-band model algorithms to retrieve Chl-*a*. Our results showed that ISE-PLS using both the water-leaving reflectance (*R*_L_) and the first derivative reflectance (FDR) had a better predictive ability with higher coefficient of determination (*R*^2^), lower root mean squared error (RMSE), and higher residual predictive deviation (RPD) values (*R*^2^ = 0.77, RMSE = 1.47 and RPD = 2.1 for *R*_L_; *R*^2^ = 0.78, RMSE = 1.45 and RPD = 2.13 for FDR). However, in this study the ocean chlorophyll (OC) algorithms had poor predictive ability and the three-band and two-band model algorithms did not perform well in areas with lower Chl-*a* concentrations. These results support ISE-PLS as a potential coastal water quality assessment method using hyperspectral measurements.

## 1. Introduction

The Seto Inland Sea is an approximately 23,000 km^2^ semi-enclosed coastal sea in western Japan, with an average depth of 38 m. This sea is well-known as one of the world’s most productive fisheries due to its abundance of fish and variety of fish species [1]. Approximately 35 million people live around the Seto Inland Sea, bringing increased industrialization and urbanization that have made the Seto Inland Sea one of Japan’s most industrialized regions [2]. However, productivity of fisheries is sensitive and thus vulnerable to anthropogenic stress. Eutrophication of coastal waters has affected fishing and other activities by contributing to harmful algal blooms (HABs), also known as red tides. HABs frequently occurred in the Seto Inland Sea during a period of high economic growth in the 1970s [3]. Although HABs have decreased from about 300 cases per year in 1976 to about 100 cases per year more recently [4], severe damage to fisheries and significant economic losses due to HABs are still occurring [5]. Therefore, monitoring HABs is vital for managing the fisheries industry and ensuring sea water quality.

The scientific community and various agencies monitor HABs to manage and control them. Many studies have conducted HABs observation, but most have relied on conventional in situ ship surveys and buoy stations [6]. For each observation, water samples must be collected and analyzed under controlled lab environments, which is costly and time consuming. Additionally, the spatial scale of such surveys is limited. Remote sensing has been widely applied to monitor, in real time, various ocean environment factors on a large scale using spaceborne or airborne instruments. Having a high degree of spatial and temporal coverage over a large scale is convenient for monitoring HABs. Several studies have shown distributions of HABs using satellite imagery and chlorophyll *a* (Chl-*a*) concentration measurements [7,8]. Chl-*a* concentration in water is a major indicator of a trophic state and oceanic Chl-*a* concentration is the most common property characterizing first trophic levels in marine environments [9]. Chl-*a* acts as a link between nutrient concentration and algal production [10], therefore, it can be used as a proxy to evaluate HABs.

Earlier studies indicated bio-optical models were usually used to estimate Chl-*a* concentrations in water [11], which are based on the inherent optical properties (IOPs) in a water body, that is, the absorption and backscattering dominated by Chl-*a*, colored dissolved organic matter (CDOM), and suspended matters etc. Chl-*a* can be estimated when the relationship between IOPs and the remote sensing reflectance (*R*_rs_) is built. Basing on the bio-optical properties, several algorithms developed to estimate Chl-*a* in the open sea and coastal area, such as the ocean chlorophyll 2-band (OC2), ocean chlorophyll 3-band (OC3), and ocean chlorophyll 4-band (OC4) algorithms used for the standard Sea-viewing Wide Field-of-view Sensor (SeaWiFS) Chl-*a* product [12,13]. The strong Chl-*a* absorption in red bands and diminishing Chl-*a* absorption and increasing water absorption in near infrared (NIR) bands [14] yields a band ratio between the NIR and red bands that has frequently been used to estimate Chl-*a* concentrations [15,16,17]. Han and Rundquist [18] found that the ratio of reflectance at 705 nm (NIR) to reflectance at 670 nm (red) correlated well with Chl-*a* concentration in a turbid reservoir. Additionally, a three-band semi-analytical reflectance model can be used to assess Chl-*a* concentration by taking advantage of the red and NIR regions’ absorption characteristics. This model was originally developed to estimate pigment content in higher plant leaves [19].

Partial least squares (PLS) regression, a statistical method developed by Wold [20], is an efficient tool for multivariate modeling that is increasingly used to handle high-dimensional hyperspectral data [21,22]. Its potential application for water quality quantification has been tested [23], and the iterative stepwise elimination PLS (ISE-PLS) [24], which combines PLS and a wavelength selection function, has proven effective at estimating Chl-*a* in inland irrigation ponds water [25]. However, ISE-PLS has not been tested in coastal waters or compared with traditional algorithms.

Our objectives are: (1) to develop models to estimate Chl-*a* using in situ hyperspectral data; (2) to evaluate traditional empirical and semi-analytical algorithms in the Seto Inland Sea; and (3) to evaluate the ISE-PLS method’s accuracy in coastal waters.

## 2. Materials and Methods

### 2.1. Study Area

**Figure 1:**
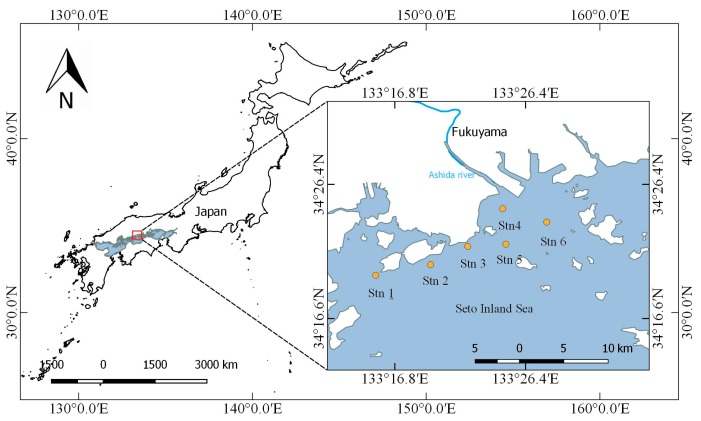
Research site locations in this study.

The study area is in the central part of the Seto Inland Sea near the city of Fukuyama as shown in Figure 1. We selected six sites as sampling stations, which are described in Table 1. The study area has an average water depth of 17.3 m and water temperatures range from 7.3 °C (winter) to 28.4 °C (summer). The Seto Inland Sea is rich in fishery resources, with more than 50% of the total fish production contributed by aquaculture production. Additionally, there are approximately 17 fish farms being operated in the Tashima and Yokota areas near our study area [26]. Consequently, there are a range of organic compounds released by fish farm waste that may affect eutrophication due to dissolved nitrogen [27].

### 2.2. Data Collection and Pre-Processing

We conducted 12 ship-based surveys between 16 December 2015, and 7 September 2017 at six stations (Figure 1). We performed in situ measurements of water-leaving reflectance (*R*_L_) using a MS-720 (Eiko Co. Ltd., Tokyo, Japan) spectrometer, with a spectral range of 350–1050 nm and a spectral interval of 3.3 nm. We interpolated the spectral interval to 1 nm when exporting data. We gathered spectral readings approximately 1 m above the water surface, with a probe field angle of 25°, between 9:00 a.m. and 11:00 a.m. under clear sky conditions. We measured Chl-*a* using a Hydrolab DS5 (Hach, Loveland, CO, USA) multiparameter data sonde with sensors for measuring Chl-*a* (range from 0 to 500 µg/L) and other water quality parameters (e.g., temperature, salinity, dissolved oxygen, etc.). In this study we used Chl-*a* data from just beneath the water surface.

With respect to spectral data, we identified reflectance ranges of 325–399 nm and 901–1075 nm as noise and removed them. We then smoothed the spectral data using a Savitzky-Golay filter with 15 smoothing points. To compare the performance for Chl-*a* estimation with the original water-leaving reflectance, we also computed the first derivative reflectance (FDR), which is calculated as the difference of water-leaving reflectance for two adjacent wavebands.

### 2.3. OC Algorithms

In this study we used the newest ocean chlorophyll (OC) algorithms (version 6) [28], which was formulated as a fourth-order polynomial with five coefficients. The newest OC algorithms yielded better statistical agreement between model data and Chl-*a* than the first version OC algorithm [12], which was a modified cubic polynomial relationship between Chl-*a* and a ratio of remote sensing reflectance *R*_rs_.

Remote sensing reflectance *R*_rs_(λ) can be represented by the relationship with water-leaving reflectance *R*_L_(λ) as follows [29]:

(1) $$R_{\mathrm{rs}}\left(\lambda \right)=\pi R_{L}\left(\lambda \right)$$

where λ is wavelength. The version 6 OC algorithms use a fourth-order polynomial equation that can be written as:

(2) $$\log_{10}\left(\mathrm{Chl}\_a\right)=a_{0}+a_{1}R+a_{2}R^{2}+a_{3}R^{3}+a_{4}R^{4}\;$$

where *R* = log_10_(*R*_rs_(λ_1_)/*R*_rs_(λ_2_)) and the coefficients *a*_0_, *a*_1_, *a*_2_, *a*_3,_ and *a*_4_ are different in the OC2, OC3, and OC4 algorithms. OC2 uses the blue/green ratio *R*_rs_ (blue)/*R*_rs_ (green) and *R* is described as *R* = log_10_ ($\frac{R_{\mathrm{rs}}\;\left(\mathrm{blue}\right)}{R_{\mathrm{rs}}\;\left(\mathrm{green}\right)})$. OC3 uses a three-band formulation with a maximum of *R*_rs_ band ratios *R*_rs_ (blue1)/*R*_rs_ (green) and *R*_rs_ (blue2)/*R*_rs_ (green), and *R* is expressed as *R* = log_10_ ($\frac{R_{\mathrm{rs}}\;\left(\mathrm{blue}1\right)\;>\;R_{\mathrm{rs}}\;\left(\mathrm{blue}2\right)}{R_{\mathrm{rs}}\;\left(\mathrm{green}\right)})$. Similarly, OC4 uses the maximum of three *R*_rs_ ratios *R*_rs_ (blue1)/*R*_rs_ (green), *R*_rs_ (blue2)/*R*_rs_ (green) and *R*_rs_ (blue3)/*R*_rs_ (green)—to build the formulation, with *R* expressed as *R* = log_10_ ($\frac{R_{\mathrm{rs}}\;\left(\mathrm{blue}1\right)\;>\;R_{\mathrm{rs}}\;\left(\mathrm{blue}2\right)\;>\;R_{\mathrm{rs}}\;\left(\mathrm{blue}3\right)}{R_{\mathrm{rs}}\;\left(\mathrm{green}\right)}$). The OC4 algorithm has been considered a standard method for satellite detection of HABs over global waters [30,31].

In view of the sensor differences between the hyperspectral spectrometer used for in situ measurements and the SeaWiFS satellite sensor, we recalculated the parameters for Equation (2) by model recalibration using in situ Chl-*a* and *R*_rs_, which was in accordance with specified OC algorithm wavebands.

### 2.4. Three-Band Model

The three-band model uses the NIR and red wavebands and is formulated as [19,32]:

(3) $$\mathrm{Chl}-a\;\propto \left(R_{\lambda 1}^{-1}-R_{\lambda 2}^{-1}\right)\;\times R_{\lambda 3}$$

where *R*_λi_ is the reflectance at a wavelength of λi nm. Previous study found the optimal spectral ranges for these wavelengths to be, λ1 = 660–670 nm, which is maximally sensitive to absorption by Chl-*a*; λ2 = 690–720 nm, which is minimally sensitive to absorption by Chl-*a*; and λ3 = 720–750 nm, which is minimally affected by absorption by any constituents (e.g., Chl-*a*, suspended solids, etc.) [32,33]. We expected to find the optimal spectral ranges of λ1, λ2, and λ3 for Chl-*a* estimation by spectrally tuning the conceptual model using a stepwise technique [34]. First, we set λ2 and λ3 to 700 nm and 750 nm, respectively, and then linearly regressed using all available bands and Chl-*a* to obtain the first estimate of λ1, with which there was a high correlation coefficient (*r*). After we fixed λ1, we set λ2 as an unknown waveband and linearly regressed to find an optimal λ2 based on the best *r* value using the reflectance corresponding to a fixed λ1 and an assumed λ3. Analogously, we confirmed the optimal λ3 using the reflectance corresponding with fixed λ1 and λ2 values.

### 2.5. Two-Band Model

The NIR/red two-band model has been widely used to retrieve Chl-*a* concentrations in turbid productive waters to identify phytoplankton blooms [35]. This model is formulated as follows:

(4) $$\mathrm{Chl}-a\;\propto \;R_{\lambda 1}^{-1}\;\times \;R_{\lambda 2}$$

where λ1 is in the red region and λ2 is in NIR region. According to the band tuning method [34], we tuned the model to select the optimal NIR and red bands for Chl-*a* retrieval in this research area and compared its accuracy with the previous model, i.e., NIR/red model using wavelengths of 705 nm in the NIR region and 670 nm in the red region [18].

### 2.6. ISE-PLS

Partial least square (PLS) is useful for handling many descriptors even when co-linearity and noise in the model building regression are present [36]. The standard PLS regression equation can be expressed as follows:

(5) $$y=\beta_{1}x_{1}+\beta_{2}x_{2}+\ldots +\beta_{i}x_{i}+\varepsilon$$

where *y* is the response variable that represents Chl-*a*, *x_i_* is the predictor variable representing spectral data such as *R*_L_ or FDR values for spectral bands 1 to *i* (400–900 nm), *β_i_* is the estimated weighted regression coefficient, and *ε* is the error vector. In the PLS model, the original predictor variables (*x*) are projected onto a small number of orthogonal latent variables to simplify their relationships with response variables (*y*) [37]. We selected the optimal number of latent variables (NLV) in the final model using the leave-one-out (LOO) cross-validation method with a minimum value of the root mean squared error (RMSE), which is calculated as follows:

(6) $$\mathrm{RMSE}=\sqrt{\frac{\sum_{i\;=\;1}^{n}{\left(y_{i\;}-\;y_{p}\right)}^{2}}{n}}$$

where *y_i_* and *y_p_* represent sample i’s measured and predicted Chl-*a*, respectively, and *n* is the number of samples in the dataset (*n* = 59).

The iterative stepwise elimination PLS (ISE-PLS) uses a model-wise elimination technique [24] that permits the removal of less useful descriptors to improve predictive performance. This process is based on the importance of the predictor *z_i_*, which is defined as:

(7) $$z_{i}=\;\frac{\left|\beta_{i}\right|s_{i}}{\sum_{i\;=\;1}^{I}\left|\beta_{i}\right|s_{i}}$$

where *I* is the maximum number of variables, *s_i_* is the standard deviation of predictor x*_i_* (each predictor includes 59 samples). PLS modeling uses all available wavebands (501 bands between 400 and 900 nm). Predictors are then evaluated based on the value of the importance of predictor *z_i_*. The predictor with minimum importance (i.e., the minimum *z_i_*) is eliminated in each elimination cycle and the remaining predictors are used to recalibrate the model [38]. Finally, a model with maximum predictive ability is selected using the minimum RMSE value from the cross-validation.

### 2.7. Evaluation of Predictive Ability

We used the coefficient of determination (*R*^2^), RMSE, and bias to evaluate the predictive ability of empirical and semi-analytical algorithms such as OC, three-band, and two-band model algorithms. Higher *R*^2^ values and a lower RMSE indicate better Chl-*a* estimation performance, and bias represents systematic difference between actual and predicted values. To evaluate the ISE-PLS predictive ability, we used *R*^2^ and RMSE from the LOO cross-validation in the final model. Additionally, we introduced the residual predictive deviation (RPD), which is defined as the ratio of the standard error of the prediction to the standard deviation, as the evaluating indicator. RPD can be expressed as RPD = SD/RMSE [39]. As shown in a previous study by Chang and Laird (2002) [40], an RPD > 2 indicates a model with good predictive ability, 1.4 < RPD < 2 indicates moderately good model in need of some improvement, and an RPD < 1.4 means the model has poor predictive ability. Finally, a method for NLV selection was used in the final PLS model, which has been reported can lower the risk of over-fitting [41]. The evaluation was basing on the sum of RMSE in cross-validation and Jaggedness (*J*), defined as:

(8) $$J_{j}=\;\sum_{i=2}^{I}\sqrt{{\left(\beta_{ji}-\beta_{ji-1}\right)}^{2}}$$

where *j* is NLV and *β_ji_* represents the regression coefficient when using *j* latent variables. At first, the RMSE in cross-validation and *J* were calculated from each NLV model (maximum of 10 latent variables were set) basing on ISE-PLS selected variables, then each value was rescaled to the range [0–1] as follows:

(9) $${\mathrm{RMSE}}_{r}=\;\frac{\mathrm{RMSE}-{\mathrm{RMSE}}_{\min}}{{\mathrm{RMSE}}_{\max}-{\mathrm{RMSE}}_{\min}}$$

(10) $$\;J_{r}=\;\frac{J-J_{\min}}{J_{\max}-J_{\min}}$$

where the subscript r refers to rescaled. The lowest value of RMSE_r_ + *J*_r_ indicates the optimal NLV for the final PLS model.

We performed all data handling and regression analyses using Matlab software version 8.6 (MathWorks, Sherborn, MA, USA).

## 3. Results

### 3.1. Chl-a Characteristics and Spectral Data

Table 2 shows Chl-*a* concentration descriptive statistics from this study, including stations, number of samples, minimum (Min), maximum (Max), mean, standard deviation (SD) and coefficient of variation (CV).

### 3.2. Comparison of Empirical and Semi-Analytical Models

#### 3.2.1. Performance of Models for All Dataset

**Figure 2:**
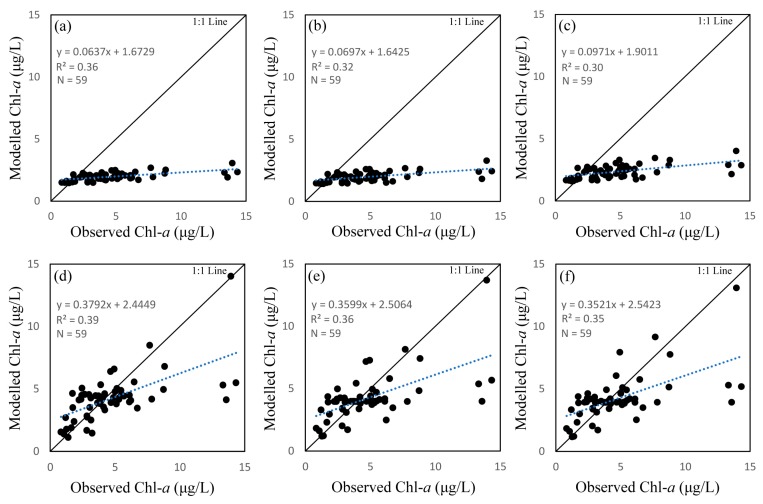
Correlation between observed and modelled chlorophyll *a* (Chl-*a*) using ocean chlorophyll (OC) algorithms. The first row shows results using standard coefficients (**a**) OC2; (**b**) OC3; and (**c**) OC4. The second row shows results from recalibrated OC models using the dataset of this study (**d**) OC2; (**e**) OC3; and (**f**) (OC4).

We used several empirical and semi-analytical models for Chl-*a* retrieval, the results of which are shown in Table 3. We initially used three standard empirical algorithms, OC2, OC3, and OC4. The first row of Figure 2 shows scatter plots between in situ measured Chl-*a* and Chl-*a* derived from OC models. The results show a linear relationship between measured and modelled Chl-*a* for all three OC algorithms, with poor *R*^2^ values (0.36, 0.31, and 0.30, respectively for OC2, OC3, and OC4). In addition, results of all three OC algorithms underestimate Chl-*a*, indicated by the bias (−2.32, −2.71, and −2.70, respectively for OC2, OC3, and OC4). The second row of Figure 2 shows scatter plots between recalibrated OC algorithms and Chl-*a*. The *R*^2^ values for all three OC algorithms were slightly improved (0.39, 0.36, and 0.35 respectively for OC2, OC3, and OC4), and scattered points were closer to the 1:1 line with a smaller bias (−0.46, −0.49, and −0.49, respectively for OC2, OC3, and OC4). For both the standard and recalibrated OC models, the OC2 algorithm performed better than OC3 and OC4 for Chl-*a* retrieval in this study; however, its predictive ability remains poor due to its low *R*^2^ value.

**Figure 3:**
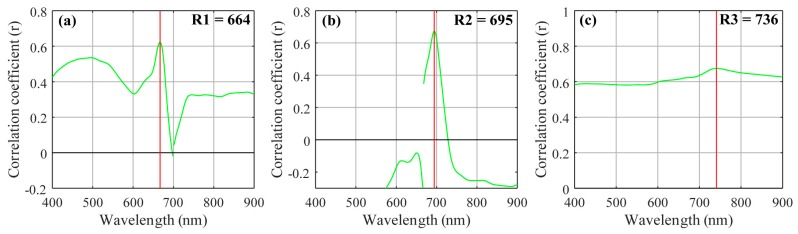
Selected wavebands for the three-band model algorithm using the tuning method (**a**) optimal band λ1; (**b**) optimal band λ2; and (**c**) optimal band λ3.

**Figure 4:**
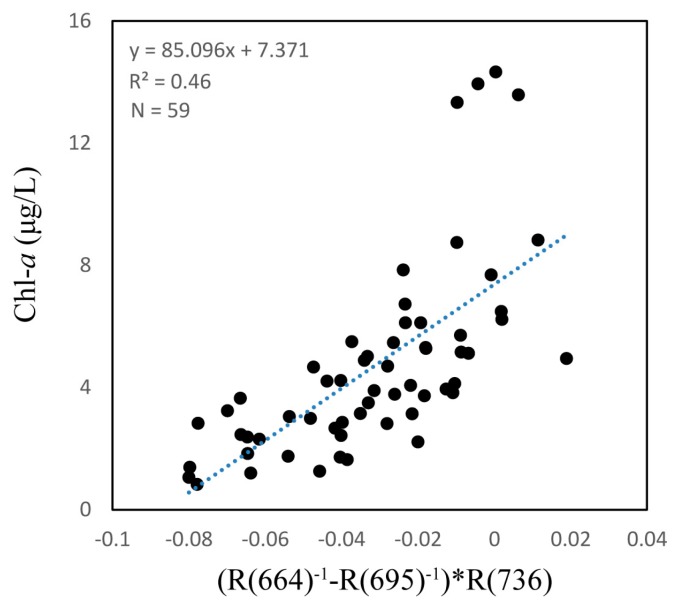
Correlation between the three-band model algorithm and measured chlorophyll *a* (Chl-*a*).

The three-band and two-band algorithms were both based on the NIR region, which has high absorption by water, and the red region, which has high absorption by Chl-*a*. Figure 3 shows the three-band algorithm tuning process. The optimal λ1 appeared at 664 nm where the *r* value is highest when using assumed λ2 and λ3 values of 700 nm and 750 nm, respectively. λ2 and λ3 appeared at 695 nm and 736 nm when using the tuning method. These results showed a linear relationship between the three-band algorithm and Chl-*a* concentration with a *R*^2^ value of 0.46, as shown in Figure 4.

**Figure 5:**
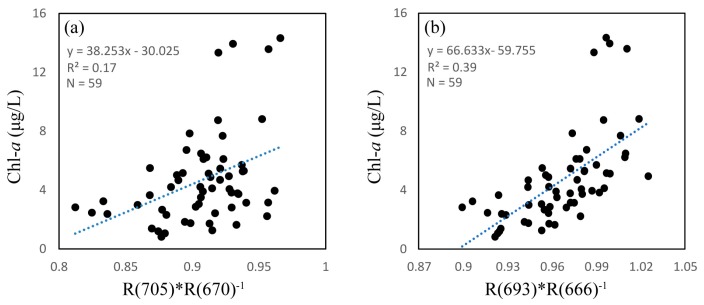
Relationship between observed chlorophyll *a* (Chl-*a*) concentration and near-infrared/red reflectance ratio, (**a**) Ratio of R(705) to R(670); (**b**) Ratio of R(693) to R(666).

**Figure 6:**
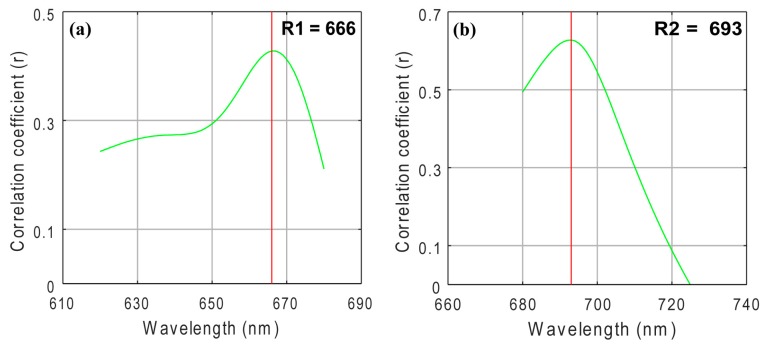
Selected wavebands for near-infrared/red algorithm using the tuning method (**a**) optimal band λ1; (**b**) optimal band λ2.

The two-band NIR red model results showed an incompact linear relationship between the reflectance ratios of 705 nm and 670 nm and measured Chl-*a*, with a poor *R*^2^ value of 0.17, as shown in Figure 5a. As with the three-band model, we tuned the spectral position to obtain the optimal NIR and red wavebands. We initially set the NIR waveband to 705 nm and then selected the optimal red region waveband, which we set from 620 nm to 680 nm based on the highest *r* value. Figure 6a shows that 666 nm was the optimal red waveband with a *r* value of 0.43. After fixing the optimal red waveband, we selected the optimal NIR region waveband, which we set from 680 nm to 740 nm. As shown in Figure 6b, we selected 693 nm as the best NIR waveband with a *r* value of 0.63. Figure 5b shows a linear relationship between the reflectance ratios of 693 nm and 666 nm and measured Chl-*a*, with a *R*^2^ of 0.39.

#### 3.2.2. Performance of Models for Separated Dataset

**Figure 7:**
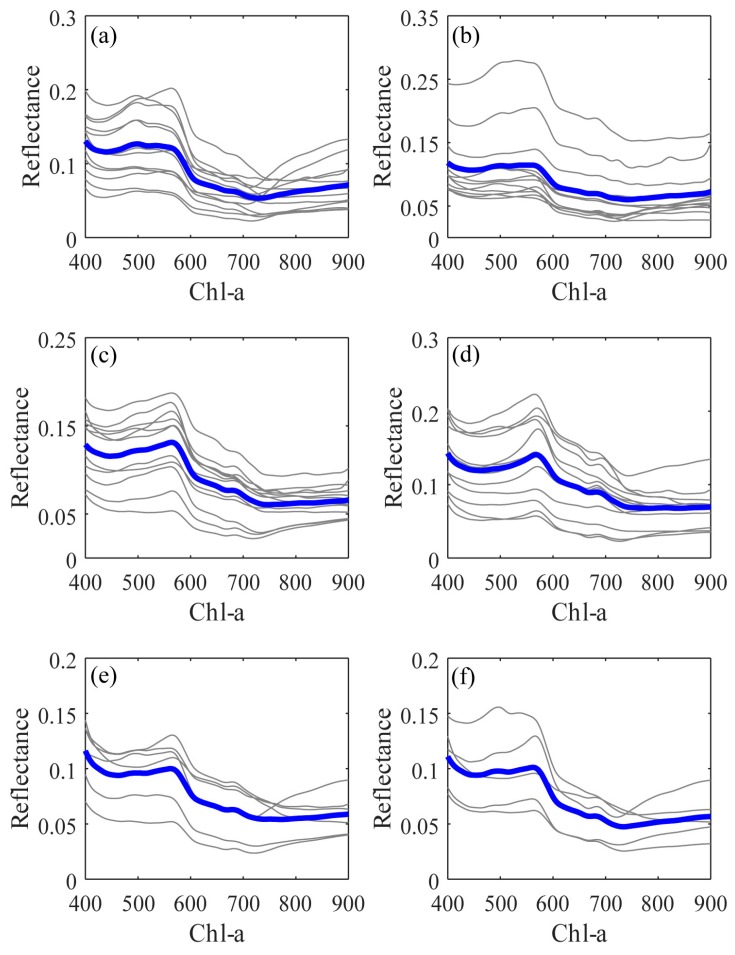
Water-leaving reflectance (*R*_L_) spectra with the spectra average (blue line) for each station ((**a**–**f**) are stations 1 to 6 in turn).

Remote sensing methods for retrieving water quality parameters contain spatial and temporal variations because the water body components that affect reflection properties vary in space and time. To further clarify the most fitted Chl-*a* retrieval method in the research area, we analysed algorithms using a separated dataset of six stations. Figure 7 shows the *R*_L_ and average *R*_L_ of each station for the research period. As we can see, the average *R*_L_ of each station shows little difference those of the others. Especially at station 4 (Figure 7d), there is an obvious reflectance peak around 580 nm, which is a result of minimum absorption by all pigments [42]. Figure 1 shows that station 4 is near a river, which could bring various nutrients from land to the coastal area. Consequently, the highest max Chl-*a* and SD values were obtained at station 4, as shown in Table 2.

**Figure 8:**
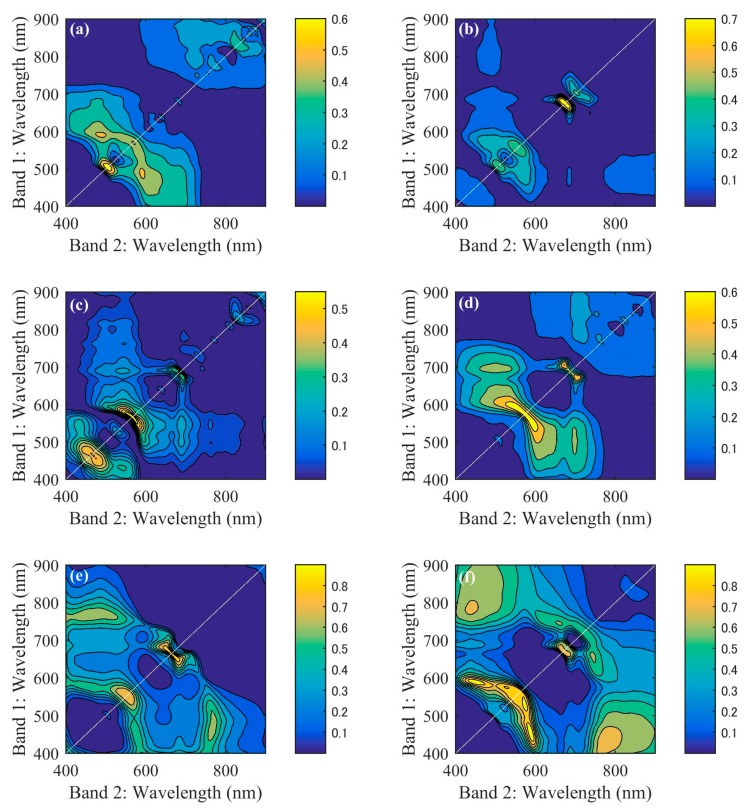
Two-dimensional *R*^2^ distributions obtained through sequential regressions using all band ratios and chlorophyll *a* (Chl-*a*) concentrations for each station ((**a**–**f**) are stations 1 to 6 in turn).

We analysed regressions using all possible band ratios in the 400 to 900 nm range and analysed Chl-*a* concentration for each station, as shown in Figure 8. Two-dimensional correlation matrixes indicate the *R*^2^ distribution for all band ratios (250,000 combinations). The yellow regions indicate high *R*^2^ values for calibration between band ratios and Chl-*a* concentration, with most figures indicating that high *R*^2^ values appear in the NIR and red regions (near 680–710 nm) and green region (near 500–600 nm). However, Figure 8a shows no correlation between NIR/red ratio and Chl-*a* concentration, which may indicate that the NIR/red ratio doesn’t fit for water areas with lower and narrower Chl-*a* concentration ranges, as indicated the lowest mean and SD values shown in Table 2, which is consistent with a previous study on band ratio analysis [18].

**Figure 9:**
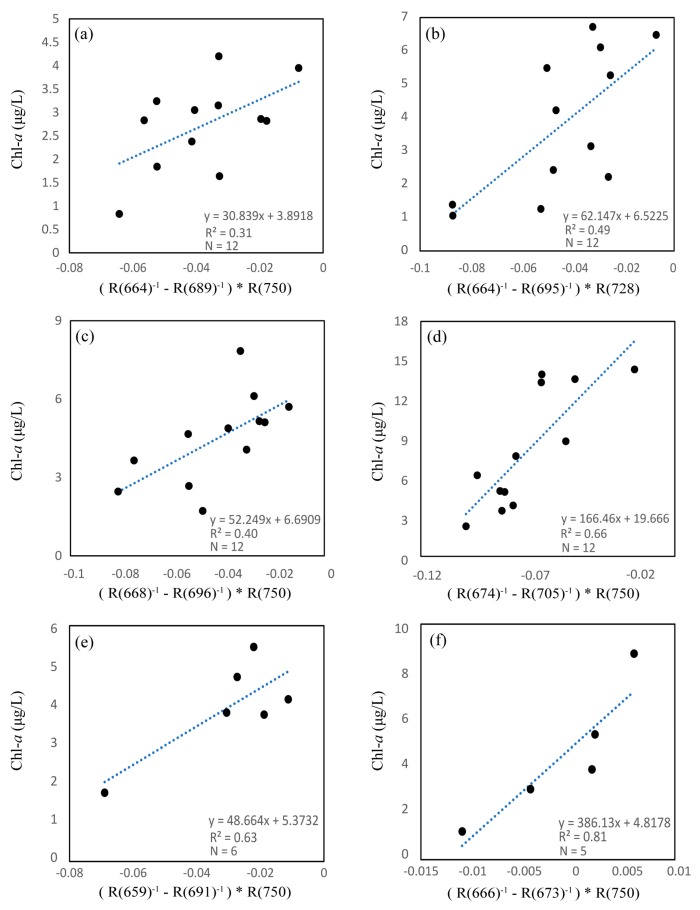
Calibrations between the three-band model algorithm and chlorophyll a (Chl-a) concentrations ((**a**–**f**) are stations 1 to 6 in turn).

We conducted calibrations between the three-band algorithm and Chl-*a* concentration at each station, the results of which are shown in Figure 9. We selected three optimal wavebands using a tuning method before conducting calibration for each station. It is apparent that station 4 performed better than other stations (1, 2, and 3) with the same dataset number (*N* = 12), with a *R*^2^ value of 0.66, using wavebands of 674, 705, and 750 nm. However, we obtained a poor *R*^2^ at station 1, which had the lowest Chl-*a* concentration in this study, using wavebands of 664, 689, and 750 nm. These results may indicate that the three-band algorithm performs well in water with relatively higher Chl-*a* concentrations, which is consistent with several previous studies [19,22]. Figure 9e,f also show better *R*^2^ values (0.63 at station 5 and 0.81 at station 6) with calibration between the three-band algorithm and Chl-*a* concentration. This provides a possibility of using the three-band algorithm to estimate Chl-*a* in these areas; nevertheless, a shortness of data (*N* = 6 at station 5, *N* = 5 at station 6) may also provide uncertainty to the results.

### 3.3. ISE-PLS Calibration and Validation

**Figure 10:**
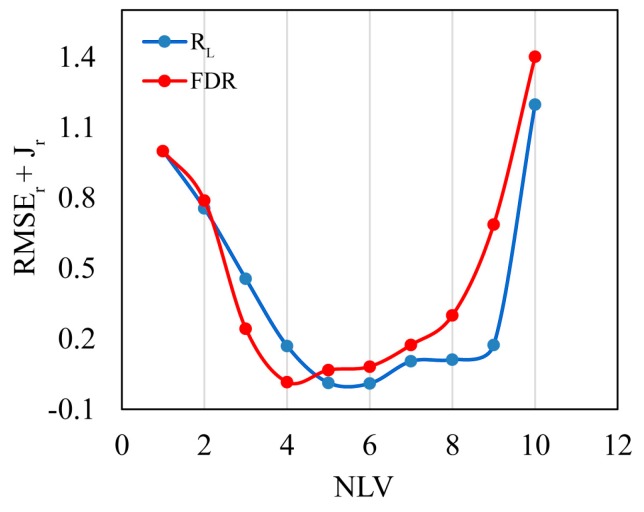
Results for the RMSE_r_ + *J*_r_ when using different number of latent variables (NLV).

**Figure 11:**
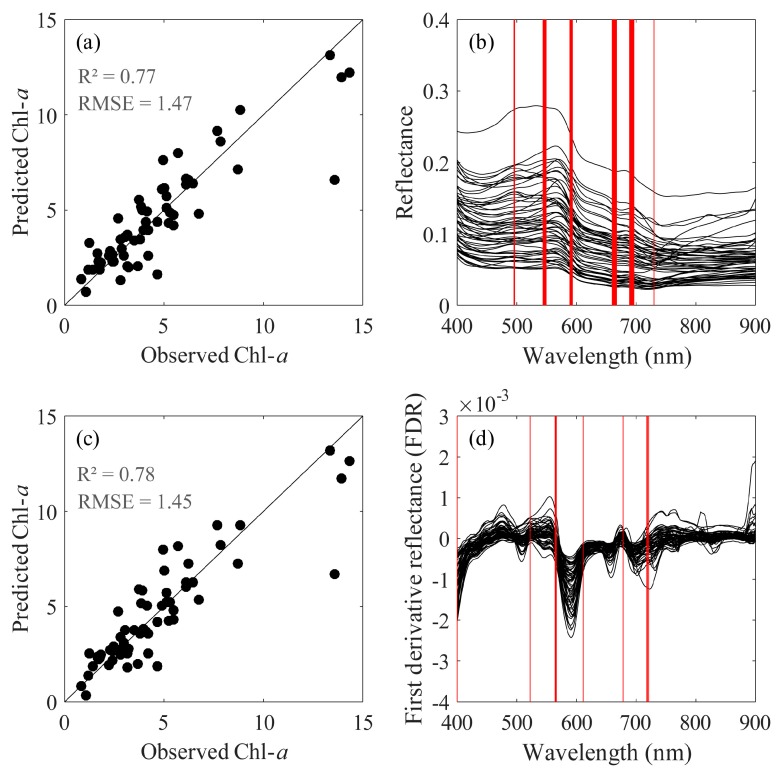
Relationship between observed and predicted chlorophyll *a* (Chl-*a*) (**a**) water-leaving reflectance (*R*_L_); (**c**) first derivative reflectance (FDR), and selected wavebands by iterative stepwise elimination partial least squares (ISE-PLS) for Chl-*a* retrieval (**b**) *R*_L_; (**d**) FDR.

ISE-PLS selected optimal variables both for *R*_L_ and FDR data due to the iterative stepwise elimination function, to select the optimal NLV in the final model, the relationship between RMSE_r_ + *J*_r_ and NLV (from 1 to 10) were analysed, as shown in Figure 10. The minimum value showed the optimal NLV (6 for *R*_L_ and 4 for FDR), larger NLV indicated over-fitting and lower NLV indicated under-fitting. Table 4 summarizes ISE-PLS calibration and validation results using *R*_L_ and FDR for Chl-*a* retrieval. As Table 4 shows, ISE-PLS had the same *R*^2^ values (0.83 for both *R*_L_ and FDR) and slightly different RMSE values (1.29 for *R*_L_ and 1.28 for FDR) for calibration. We also found that ISE-PLS using both datasets had better Chl-*a* retrieval performance than other algorithms, which was indicated by *R*^2^ (0.77 for *R*_L_ and 0.78 for FDR) and RPD (2.10 for *R*_L_ and 2.13 for FDR) values in the validation results. ISE-PLS using FDR performed marginally better than ISE-PLS using *R*_L_ because of the higher *R*^2^ and RPD and lower RMSE (1.47 for *R*_L_ and 1.45 for FDR) values for validation. Figure 11a,c show validation plots for ISE-PLS using *R*_L_ and FDR, respectively. Both figures show a close linear relationship between predicted and observed Chl-*a* with the exception of a few scatter points.

Because of the iterative stepwise elimination function, we selected the optimal wavebands using ISE-PLS for both *R*_L_ and FDR datasets based on the lowest RMSE for validation, as shown in Figure 11b,d. Selected wavebands for *R*_L_ ranged from 495 to 496 nm [43], 589 to 593 nm [44], and 660 to 667 nm [45], which had been proven related to phytoplankton absorption, 544 to 549 nm [43], and 689 to 696 nm [32], which indicated relationship with Chl-*a* fluorescence, and 730 nm which is also sometimes used for Chl-*a* retrieval [45]. We selected a total of 30 (6%) informative wavebands from all 501 wavebands. And for FDR, we selected 10 (2%) informative wavebands from all 501 wavebands.

**Figure 12:**
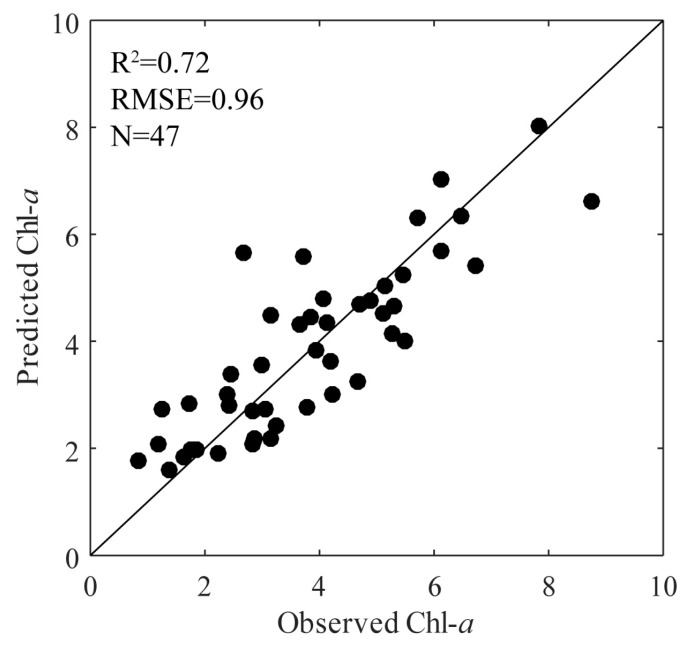
Validation of iterative stepwise elimination partial least squares (ISE-PLS) method using lower chlorophyll *a* (Chl-*a*) concentration dataset (station 4 not included).

In this study, station 4 may have been affected by river nutrients, we carried out ISE-PLS regressions using the *R*_L_ dataset except for at station 4 to decrease the impact of different water types. Figure 12 shows the validation plot between observed and predicted Chl-*a*, which was obtained using the LOO method in the ISE-PLS regression. As we can see, the maximum Chl-*a* concentration (from 14.33 to 8.74 μg/L) decreased after removing the station 4 dataset. Results shows a close linear relationship between observed and predicted Chl-*a*; however, compared to the *R*^2^ value (0.77) obtained by ISE-PLS validation using all datasets (*N* = 59), a relatively lower *R*^2^ value (0.72) was obtained using the datasets except station 4 (*N* = 47), which may indicate that ISE-PLS performed better in water areas with a wide range of Chl-*a* concentrations.

## 4. Discussion

### 4.1. Empirical and Semi-Analytical Models Performance

In the present study, several empirical and semi-analytical models have been established for Chl-*a* estimation in the Seto Inland Sea. Results show all the standard empirical OC algorithms have underestimated the in situ Chl-*a*, which turns out to be lower RMSE values. The underestimated Chl-*a* calculated from standard satellite algorithms possibly due to the uncertainty in the performance of atmospheric-correction algorithms [46]. The coefficients for OC algorithms have been recalculated using in situ Chl-*a* and hyperspectral dataset, however, OC algorithms using recalculated coefficients also show lower *R*^2^ and higher RMSE values. The three-band model utilized in this study shows a better *R*^2^ than all OC algorithms, and the optimal spectral bands (664, 695, and 736 nm) selected from band tuning are in accord with previous study [19]. However, the low accuracy may indicate the model is unstable as the RMSE shown, which may attribute to the lower Chl-*a* concentration (maximum value of 14.33 μg/L) in this study, because other optically active constituents (CDOM, tripton etc.) may affect the water reflection properties [47]. Similarly, the NIR/red tuning model also shows a better *R*^2^ than OC algorithms, while a poor accuracy, the optimal spectral bands (666 and 693 nm) are in accord with previous study [19]. For the NIR/red model using bands 670 and 705 nm, result shows a poor accuracy, which may attribute to the red shift property [48], that is, there is a red shift in the fluorescence peak to the longer wavelength in higher Chl-*a* concentration waters.

### 4.2. ISE-PLS Performance

Through the above analyses, it has been shown that ISE-PLS using both *R*_L_ and FDR performed better than other algorithms, including the OC, three-band model, and NIR/red two-band model algorithms, indicated by higher *R*^2^ and lower RMSE values. This finding is consistent with previous studies in which PLS method can be used as useful method in retrieval of water quality parameters [22,25]. In addition, ISE-PLS using FDR performed better than ISE-PLS using *R*_L_ as indicated by higher *R*^2^ and RPD values and lower RMSE for validation. This may have resulted from derivative analysis reducing random noise and removing the effects of suspended matter on Chl-*a* concentration estimates [22]. Using ISE-PLS, a total of 30 (6%) wavebands for *R*_L_ and 10 (2%) wavebands for FDR have been selected as optimal bands from all 501 wavebands, separately, which indicate that 94% wavebands when using *R*_L_ and 98% wavebands when using FDR are redundant for Chl-*a* estimation in the Seto Inland Sea. This result provide an evidence that ISE-PLS can be used as an approach for optimal wavebands selection, especially when using hundreds wavebands of hyperspectral data.

### 4.3. Applications of ISE-PLS Method

This study established a potential model for Chl-*a* estimation in the Seto Inland Sea, which provide the possibility for detecting HABs, since Chl-*a* concentrations can be used as indices of HABs [30]. In general, HABs detection using Chl-*a* algorithms involve the generalized relationship between a high chlorophyll content and HABs occurrences [6]. However, due to the sampling data show lower Chl-*a* concentrations and short of HABs occurrences data, it is difficult to build the relationship between HABs and Chl-*a* concentrations in this study. Nevertheless, the ISE-PLS is a useful method to estimate Chl-*a* concentration, which can be used to evaluate the water quality, so as to management the aquaculture in the Seto Inland Sea.

## 5. Conclusions

In this study, we developed various models for estimating water Chl-*a* concentration in the Seto Inland Sea, including ISE-PLS using both *R*_L_ and FDR and other methods such as OC, three-band model, and two-band model algorithms. Our results showed that the ISE-PLS method is effective for predicting Chl-*a* concentration in the Seto Inland Sea using in situ measured spectral data. With a higher prediction accuracy, ISE-PLS also selects important wavebands that match previously published studies. Additionally, ISE-PLS using FDR is marginally enhanced compared to using *R*_L_ for Chl-*a* retrieval. However, OC algorithms are not robust in this present study, and three-band and two-band model algorithms did not perform well in water areas with lower Chl-*a* concentration. Our results also indicate that the ISE-PLS method can perform better when used in water areas with a wide range of Chl-*a* concentrations. These results provide potential insights into coastal water quality assessment by using a Chl-*a* estimation method with hyperspectral measurements.

## Figures and Tables

**Table 1:** Stations for data collection in the study.

Station ID	Latitude	Longitude	Depth (m)
1	34°19′44″ N	133°15′24″ E	29
2	34°20′31″ N	133°19′26″ E	17
3	34°21′51″ N	133°22′10″ E	10
4	34°24′37″ N	133°24′44″ E	10
5	34°22′01″ N	133°24′58″ E	21
6	34°23′38″ N	133°27′58″ E	17

**Table 2:** Chl-*a* concentration (μg/L) descriptive statistics.

Stations	*N*	Min	Max	Mean	SD	CV
1	12	0.83	4.2	2.73	0.95	0.35
2	12	1.06	6.72	3.82	2.15	0.56
3	12	1.72	7.84	4.5	1.71	0.38
4	12	2.31	14.33	8.13	4.54	0.56
5	6	1.75	5.46	3.92	1.25	0.32
6	5	1.2	8.74	4.41	2.84	0.64
Total	59	0.83	14.33	4.67	3.11	0.67

*N*, number of samples; SD, standard deviation; CV, coefficient of variation.

**Table 3:** Regression models used to estimate Chl-*a* concentrations.

Algorithms	Results Equation	Bands Combination (*R*), Coefficient *a*, and Intercept *b*	*R* ^2^	RMSE	Bias
OC2	$\mathrm{Chl}\_a=10^{a_{0}+a_{1}R+a_{2}R^{2}+a_{3}R^{3}+a_{4}R^{4}}$	*R* = log_10_ ($\frac{R_{\mathrm{rs}}\left(490\right)}{R_{\mathrm{rs}}\left(555\right)}$) *a* = [0.2511 −2.0853 1.5035 −3.1747 0.3383]	0.36	3.96	−2.32
OC3	$\mathrm{Chl}\_a=10^{a_{0}+a_{1}R+a_{2}R^{2}+a_{3}R^{3}+a_{4}R^{4}}$	*R* = log_10_ ($\frac{R_{\mathrm{rs}}\left(443\right)\;>\;R_{\mathrm{rs}}\left(490\right)}{R_{\mathrm{rs}}\left(555\right)}$) *a* = [0.2515 −2.3798 1.5823 −0.6372 −0.5692]	0.32	3.95	−2.71
OC4	$\mathrm{Chl}\_a=10^{a_{0}+a_{1}R+a_{2}R^{2}+a_{3}R^{3}+a_{4}R^{4}}$	*R*= log_10_ ($\frac{R_{\mathrm{rs}}\left(443\right)\;>\;R_{\mathrm{rs}}\left(490\right)\;>\;R_{\mathrm{rs}}\left(510\right)}{R_{\mathrm{rs}}\left(555\right)}$) *a* = [0.3272 −2.9940 2.7218 −1.2259 −0.5683]	0.30	3.66	−2.70
Recalibrated OC2	$\mathrm{Chl}\_a=10^{a_{0}+a_{1}R+a_{2}R^{2}+a_{3}R^{3}+a_{4}R^{4}}$	*R* = log_10_ ($\frac{R_{\mathrm{rs}}\left(490\right)}{R_{\mathrm{rs}}\left(555\right)}$) *a* = [−8942.6 −2053.3 −100.25 −3.8257 0.5738]	0.39	2.65	−0.46
Recalibrated OC3	$\mathrm{Chl}\_a=10^{a_{0}+a_{1}R+a_{2}R^{2}+a_{3}R^{3}+a_{4}R^{4}}$	*R* = log_10_ ($\frac{R_{\mathrm{rs}}\left(443\right)\;>\;R_{\mathrm{rs}}\left(490\right)}{R_{\mathrm{rs}}\left(555\right)}$) *a* = [5204.7 −461.22 −41.033 −4.4207 0.5491]	0.36	2.50	−0.49
Recalibrated OC4	$\mathrm{Chl}\_a=10^{a_{0}+a_{1}R+a_{2}R^{2}+a_{3}R^{3}+a_{4}R^{4}}$	*R* = log_10_ ($\frac{R_{\mathrm{rs}}\left(443\right)\;>\;R_{\mathrm{rs}}\left(490\right)\;>\;R_{\mathrm{rs}}\left(510\right)}{R_{\mathrm{rs}}\left(555\right)}$) *a* = [−30610 −4098 −57.405 −0.1942 0.5933]	0.35	2.53	−0.49
Three-band	Chl_a=aR + *b*	*R* = ($R{\left(664\right)}^{-1}-R{\left(695\right)}^{-1}$) × R(736) *a* = 85.096 *b* = 7.371	0.46	2.28	3.2 × 10^−6^
NIR/red	Chl_a=aR + *b*	*R* = R(705) × $R{\left(670\right)}^{-1}$ *a* = 0.0044 *b* = 0.8863	0.17	4.88	1.8 × 10^−4^
NIR/red tuning	Chl_a=aR + *b*	*R* = R(693) × $R{\left(666\right)}^{-1}$ *a* = 66.633 *b* = −59.755	0.39	2.40	2.9 × 10^−6^

OC2, ocean chlorophyll-2; OC3, ocean chlorophyll-3; OC4, ocean chlorophyll-4; NIR, near-infrared.

**Table 4:** The coefficient of determination (*R*^2^) and root mean square error (RMSE) for calibration of iterative stepwise elimination partial least squares (ISE-PLS) and leave-one-out (LOO) cross-validation using the entire dataset (*N* = 59), with residual predictive deviation (RPD), number of wavebands, and percent ratio in the full spectrum (*i* = 501).

Dataset	*N*	Calibration			Validation			Number of Selected Wavebands	Percentage of Selected Wavebands (%)
		NLV	*R* ^2^	RMSE	*R* ^2^	RMSE	RPD		
*R*_L_	59	6	0.83	1.29	0.77	1.47	2.1	30	6.0
FDR	59	4	0.83	1.28	0.78	1.45	2.13	10	2.0

*N*, number of samples; NLV, number of latent variables; *R*_L_, water-leaving reflectance; FDR, first derivative reflectance.
